# In Silico Identification of the Laccase-Encoding Gene in the Transcriptome of the Amazon River Prawn *Macrobrachium amazonicum* (Heller, 1862)

**DOI:** 10.3390/genes15111416

**Published:** 2024-10-31

**Authors:** Gabriel Monteiro de Lima, Fernando Araújo Abrunhosa, Bruna Ramalho Maciel, Ítalo Lutz, Janieli do Socorro Amorim da Luz Sousa, Carlos Murilo Tenório Maciel, Cristiana Ramalho Maciel

**Affiliations:** Instituto de Estudos Costeiros, Campus Universitário de Bragança, Universidade Federal do Pará, Alameda Leandro Ribeiro s/n, Bragança 68600-000, PR, Brazil

**Keywords:** functional genome, hepatopancreas, lignin, lignocellulolytic fibers

## Abstract

Background: *Macrobrachium amazonicum* is an opportunistic and omnivorous species that primarily feeds on plant material. Recent studies have shown that Endo-β-1,4-glucanase and Endo-β-1,4-mannanase are expressed in the transcriptome of adult specimens, while juveniles are capable of digesting nutrients from purified cellulose in their diet. In organisms that degrade raw plant material, laccase plays a key role in oxidizing phenolic compounds found in lignin, leading to its depolymerization and increasing access to cellulose and hemicellulose microfibrils. Objective: In this study, we conducted an in silico identification and characterization of the *laccase-encoding* gene, as this enzyme is linked to lignin biodegradation in herbivorous crustaceans. Methods: We analyzed the transcriptomes of the hepatopancreas from adult *M. amazonicum*, sequenced using the Illumina HiSeq 2500 platform. Subsequently, bioinformatics analyses were conducted to predict the conserved regions and active sites associated with laccase activity. Results: A complete open reading frame (ORF) of the laccase protein was identified in all datasets, comprising 609 amino acids. The top 40 similarity hits corresponded exclusively to crustaceans such as prawns, crayfish, and crabs (86.3–51.4%), while the highest divergence was observed in relation to fungi, plants, and bacteria. Three conserved domains were detected, along with the complete set of copper-binding centers (T1Cu, T2Cu, and T3Cu). A notable variable residue was methionine, suggesting a reduced redox potential in *M. amazonicum* laccase. Conclusion: These findings, combined with recent reports on the nutritional requirements of *M. amazonicum*, contribute to a deeper understanding of the digestive physiology of this species and offer valuable insights into its ability to utilize plant fibers as energy sources.

## 1. Introduction

The Amazon River prawn *Macrobrachium amazonicum* (Heller, 1862) is a native crustacean of South America, widely distributed across the major hydrographic basins from Venezuela to Argentina, including the Orinoco, Amazon, Araguaia-Tocantins, São Francisco, and La Plata systems [1,2,3]. In northern Brazil, this species holds significant socioeconomic importance, as it is widely used in local cuisine and contributes to food security and income for many families in the Amazon region [2,4,5]. Additionally, *M. amazonicum* can account for up to 80% of the crustacean macrofauna in the turbid waters of flooded areas within the Amazon basin [6,7].

Various biological aspects of *M. amazonicum* have been extensively studied in recent years, including its digestive physiology. The Amazon River prawn is classified as an opportunistic omnivorous species, with plant material forming a significant part of its diet [8,9]. Studies of wild *M. amazonicum* populations revealed that plant debris, such as leaves, seeds, and twigs, constituted the majority of their stomach content, highlighting the importance of these items in their diet [10,11]. Furthermore, stable isotope analyses have shown that carbon from plant sources contributes to muscle growth pathways in the Amazon River prawn [12].

Additionally, studies on digestive enzymes expressed in the hepatopancreas provide valuable insights into the dietary and nutritional requirements of crustaceans, a process that has been greatly advanced by next-generation sequencing (NGS) of functional genomes [13,14,15]. Preliminary research on *M. amazonicum* identified nine fragments of digestive enzymes related to the digestion of proteins, lipids, and carbohydrates, including two enzymes involved in the breakdown of plant fibers (Endo-1,4-β-glucanase and Endo-1,4-β-mannanase) [16]. Subsequent studies confirmed the endogenous expression of Endo-1,4-β-glucanase in the Amazon River prawn [17]. Furthermore, it was observed that adding purified cellulose to the diet enhanced juvenile performance, as they utilized the plant fiber for energy, thus reducing their protein requirements. These findings raise questions about how efficiently *M. amazonicum* can digest plant fibers.

Plant fibers are primarily composed of cellulose, hemicellulose, and lignin. Cellulose and hemicellulose account for approximately 40–50% and 15–30% of the dry mass of plants, respectively, and are essential components of plant cell walls. These compounds play key roles in nutrient cycling, as their degradation releases carbon and other nutrients into the ecosystem [18]. Lignin, by contrast, comprises about 10–30% of plant mass and serves a crucial function in protecting lignocellulosic biomass. It is a heterogeneous alkyl-aromatic polymer made up of three aromatic alcohols that vary in their degree of methoxylation. Partial lignin degradation is necessary to access the cellulose and hemicellulose microfibrils. The primary enzymes involved in this process are lignin peroxidase, manganese peroxidase, and laccase [19,20,21].

Laccases are enzymes that belong to the multicopper oxidase family [22] and are well-documented in white-rot fungi, where they participate in lignin depolymerization [23,24]. These enzymes catalyze the oxidation of phenolic compounds in lignin, using molecular oxygen as the electron acceptor [19]. Laccase facilitates the oxidation of phenolic groups, generating free radicals and promoting the cleavage of chemical bonds. The enzyme typically involves three copper ion-binding sites: one mononuclear and two trinuclear centers [25]. These centers consist of 12 amino acid residues, 11 of which are highly conserved, while one is variable across different biological groups (methionine, leucine, or phenylalanine), imparting distinct redox potentials to the enzyme in fungi, bacteria, plants, and animals [26].

In crustaceans, the identification of laccase-like proteins has been linked to the immune system and oxidative stress, with methionine being noted as the variable amino acid, indicating a lower redox potential [26,27,28]. However, in the herbivorous crab *Chiromantes haematocheir*, laccase has been associated with lignin digestion, aiding cellulose access [29]. This suggests that laccase may also play a role in the digestive processes of decapods that consume plant material.

Considering the feeding habits of the Amazon River prawn and the evidence of enzymes capable of acting on cellulose and hemicellulose in its hepatopancreas, the objective of the present study was to determine whether the *laccase-encoding* gene is expressed in the functional genome of this species through in silico protein characterization. This information provides new insights into the abilities of *M. amazonicum* to digest lignin as a strategy for accessing cellulose and hemicellulose as energy sources. Furthermore, these findings are valuable for identifying the potential use of purified cellulose and other raw plant fibers in the diet of the Amazon River prawn.

## 2. Materials and Methods

The database utilized in the present study was derived from four cDNA libraries of the hepatopancreas, each created from a pool of 10 adult *M. amazonicum* specimens and sequenced using the Illumina HiSeq 2500 platform (Illumina, San Diego, CA, USA). The specimens were reared in excavated tanks at the Aquaculture Center of UNESP in Jaboticabal, São Paulo, Brazil (CAUNESP) for four months and were fed commercial pelleted feed designed for marine shrimp. These animals are descendants of a native population from the estuary of Mosqueiro Island in the coastal Amazon state of Pará, northern Brazil (01°12′37.7″ S, 46°08′17.1″ W).

After sequencing, low-quality reads (Q < 20) were evaluated and excluded using FastQC v0.12.0 [30] and Trimmomatic v0.39 [31]. Subsequently, the transcriptomes were assembled using a de novo approach, without referencing a genome, in the software Trinity v2.15.2 [32]. The search for target sequences of the laccase enzyme within the *M. amazonicum* dataset was conducted using the software MEB v0.9.2 [33] based on the identification of the most similar transcripts as determined by the BLASTn algorithm. Searches were optimized using a reference nucleotide sequence of laccase available for the land crab *C. haematocheir* (accession number LC597534.1 [29]).

The sequences identified by MEB were visualized and edited using BioEdit 7.1 [34], employing the Clustal W tool [35]. The amino acid (aa) coding regions were then inferred using ORFfinder https://www.ncbi.nlm.nih.gov/orffinder/ (accessed on: 20 November 2023). Initially, the conserved domains were reconstructed using the simple modular architecture research tool (SMART) to characterize the conserved regions of laccase in *M. amazonicum* [36]. This was followed by the identification of active sites based on literature reports and the software InterProScan 5 [37]. The molecular weight of the final protein was estimated using the ExPASy ProtParam tool [38], while peptide signal and cleavage sites were predicted using the Prop 1.0 Server [39].

Next, a search using the BLASTp tool was conducted to identify laccase-like sequences (top hits) of *M. amazonicum* in NCBI (accessed on: 6 March 2024). The available sequences of decapods were automatically aligned using Clustal Omega [40]. The outputs were then exported to Espript 3.0 [41] for the automatic identification of conserved and semiconserved protein regions. Subsequently, sequences from other crustaceans, insects, fungi, plants, and bacteria (Table 1) were incorporated into the final dataset to construct a cladogram representing the relationships between the proteins identified in *M. amazonicum* and other biological groups. The cladogram was generated using maximum likelihood based on 1000 bootstrap pseudoreplicates, employing the WAG+I+G4 evolutionary model in the software IQTREE 1.6.12 [42].

The spatial conformation of the protein was predicted based on available models of homologous crystalline structures in the protein data bank (PDB), identified using the Swiss Model [49]. The secondary structures, including major and minor α-helices, β-sheets, and disulfide bonds, were determined through multiple alignments with proteins in the PDB using ENDscript 2.0 [41]. This information was then utilized to construct the laccase conformation structure in PyMOL v2.5.7 [50].

The laccase sequence from the transcriptome of *M. amazonicum* served as a template for designing specific primers using Primer Express 3.0. Gene amplification tests were conducted in triplicate with a sample from an adult specimen of *M. amazonicum* collected in northeastern Pará (Bragança, Pará, Brazil) (1°01′49.04″ S, 46°45′14.26″ W). Total RNA was then isolated from the hepatopancreas, muscle, gills, ovaries, and male reproductive system (MRS) using the PureLink™ RNA Mini Kit (Invitrogen, Waltham, MA, USA) following the manufacturer’s instructions. The quality and integrity of the isolated RNA were assessed using a UV spectrophotometer (PicoDrop spectrophotometer; Saffron Walden, Cambridgeshire, UK) and by electrophoresis on a 1% agarose gel. The RNA sequences were converted into complementary DNA (cDNA) using the High-Capacity cDNA Reverse Transcription Kit (Invitrogen), adhering to the manufacturer’s instructions, followed by quality and integrity analysis as described above.

Genomic DNA was also isolated from the hepatopancreas and muscle of the same samples using the Wizard^®^ Genomic DNA Purification Kit (Promega, Madison, WI, USA), following the manufacturer’s instructions. The quality and integrity of the isolated DNA were evaluated as previously described, and the DNA samples were standardized to a concentration of 50 μg/μL. This procedure was performed to ascertain whether the target encoding gene is indeed present in the genome of *M. amazonicum* rather than resulting from symbiotic processes.

Ovigerous females were also collected in northeastern Pará and transported to the laboratory, where they were kept in tanks until larval hatching. Following this, *M. amazonicum* larvae were collected at different developmental stages in triplicate: larvae zoea I (LZ-I), larvae zoea V (LZ-V), larvae zoea IX (LZ-IX), post-larvae I (PL-I), post-larvae 30 (PL-30), and post-larvae 90 (PL-90) [7]. The specimens were stored until the extraction of total RNA and the synthesis of cDNA molecules, as previously described, to analyze the expression of laccase at different ontogenetic stages.

Both cDNA and DNA samples were used as templates in PCR and RT-PCR, respectively, utilizing the specific laccase primers designed for this study: Lac-F (5′-ACG ACG TCG ATT CAT TCT CC-3′) and Lac-R (5′-TGG TAG CAA GCG TGT TGT TC-3′). As a positive control, a partial fragment of the mitochondrial Cytochrome C Oxidase Subunit I (COI) gene was amplified using the primers COI-A (5′-GTA TAA GCG TCT GGG TAG TC-3′) and COI-F (5′-CCT GCA GGA GGA GGA GAY CC-3’), as described by Palumbi & Benzie [51].

The amplification reactions were conducted using the Taq DNA polymerase kit (Invitrogen, Waltham, MA, USA), comprising 1.6 μL of dNTPs, 1 μL of 10× buffer, 0.5 μL of MgCl_2_, 0.5 μL of each primer (Lac/COI), 0.13 μL of Taq DNA polymerase, 0.4 μL of template cDNA/DNA, and ultrapure water to a final volume of 10 μL. The PCR conditions included an initial denaturation step at 94 °C for 4 min, followed by 35 cycles of denaturation at 94 °C for 30 s, annealing at 57.2 °C (for Lac) or 60 °C (for COI) for 45 s and extension at 72 °C for 45 s, concluding with a final extension step at 72 °C for 5 min.

The high-quality RT-PCR products were sequenced using Sanger’s method to confirm the identification and amplification of the target fragment in the transcriptome. Initially, the samples were purified with PEG 8000 to remove any residual reagents [52] and then sequenced using the ABI Genetic Analyzer 3500 XL automatic sequencer (Applied Biosystems, Waltham, MA, USA).

## 3. Results

### 3.1. Identification of Ma-Lac

**Figure 1:**
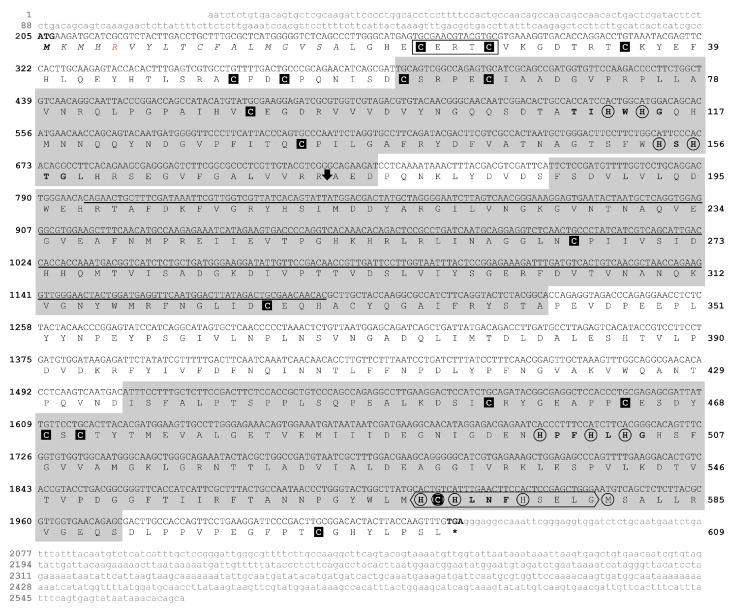
Sequences of the cDNA and the predicted protein for the laccase enzyme of *M. amazonicum*. The cDNA sequence, comprising 1830 nucleotides (nt) that translate into a protein of 609 amino acids (aa), is presented in uppercase letters. The untranslated regions (UTRs) are displayed in lowercase grey letters, consisting of 204 nt at the 5′ end and 536 nt at the 3′ end. The three multicopper oxidase domains are highlighted in gray. Conserved cysteine residues are enclosed in black squares, while the 12 copper-binding residues are marked with circles. The rectangle indicates the conserved CxRxC region characteristic of invertebrates, and the hexagon represents the Cu-oxidase binding region. The downward arrow (⬇) denotes the cleavage site between residues R_173_–A_174_. The R5 amino acid, highlighted in red, indicates the variation recorded in the R/L sequences. The underlined region signifies the amplified and sequenced gene fragment. The asterisk (*) indicates the stop codon.

The putative *laccase encoding* gene (EC 1.10.3.2) of *M. amazonicum* (Ma-Lac) was identified in the hepatopancreas across the four cDNA libraries analyzed. All fragments encompassed the complete open reading frame (ORF), comprising 1830 nucleotides and encoding a protein of 609 amino acids (Table 1). This protein is divided into two polypeptide chains (M_1_–R_173_ and A_174_–L_609_ aa) with a molecular weight of 67.7 kDa. The consensus sequence of this protein exhibited a single variable site (R–L_5_) (accession number PQ319832.1). The untranslated regions (UTRs) of the characterized sequence included 204 nucleotides at the 5’ end and 536 nucleotides at the 3’ end. The peptide signal was identified within the first 18 amino acids of the analyzed protein. Three conserved multicopper oxidase domains (Pfam Cu-oxidases) were identified, comprising 116 amino acids (C_61_–D_176_), 156 amino acids (F_187_–A_342_), and 156 amino acids (I_435_–S_590_), categorized as Pfam Cu-oxidase_3, Pfam Cu-oxidase, and Pfam Cu-oxidase_2, respectively (Figure 1).

The Ma-Lac sequences exhibited four regions typically associated with copper binding: Cu-I (T_110_IHWHG_115_), Cu-II (H_154_SHTG_158_), Cu-III (H_498_PFHLHG_504_), and Cu-IV (H_569_CHLNFH_575_). Most of these sites were highly conserved compared to laccases from other species. Notably, 12 active binding sites were identified as being involved in the formation of the mononuclear T1Cu center (H_498_, C_570_, H_575_, M_580_) and the trinuclear T2Cu/T3Cu centers (H_112_, H_501_ e H_114_, H_154_, H_156_, H_503_, H_569_, H_571_, respectively). The Cu-oxidase binding site and C_23_xRxC_27_ sites were also predicted in the protein (H_569_CHLNFHSELG_579_), which corresponds to a conserved region in the laccases of invertebrates. Additionally, 17 conserved cysteine residues were identified in *M. amazonicum* (Figure 1).

### 3.2. Alignments and Cladogram

**Figure 2:**
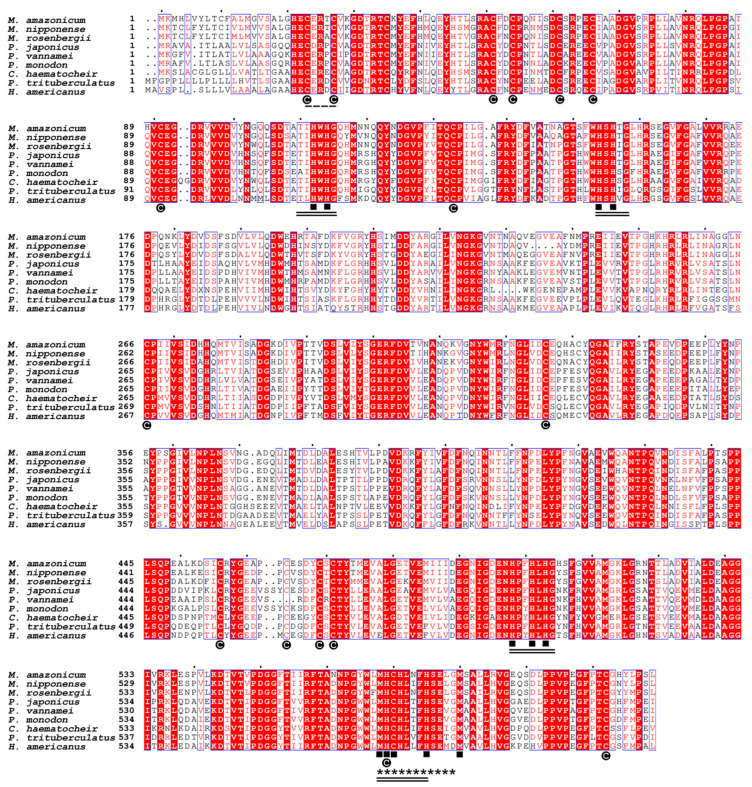
The multiple alignment of laccase enzyme sequences identified in *M. amazonicum* and other decapods available at NCBI demonstrated similarity levels ranging from 86.3% to 58.2%. Regions highlighted in red indicate fully conserved areas, while regions with a white background represent partially conserved sites. Cysteine residues (C) were found to be highly conserved, and the 12 copper-binding sites are fully conserved (■). The residues associated with Cu-I, Cu-II, Cu-III, and Cu-IV are emphasized with double lines, and the Cu-oxidase binding region is marked with set of asterisks (*). Additionally, the invertebrate-specific CxRxC domain was conserved across all species analyzed. The sequence accession codes are shown in Table 1.

The comparative analysis of the Ma-Lac amino acid sequence using the BLASTp tool against the NCBI database yielded 40 top hits, all represented by crustaceans (Table S1; accessed on 6 March 2024). Multiple alignments of the amino acid sequences indicated that Ma-Lac exhibited the highest similarity to laccases from congeneric species, *Macrobrachium nipponense* (86.3%) and *M. rosenbergii* (85.5%). In contrast, comparisons with laccase sequences from other decapods revealed similarity levels ranging from 64.1% to 58.2% (Table 1; Figure 2).

**Figure 3:**
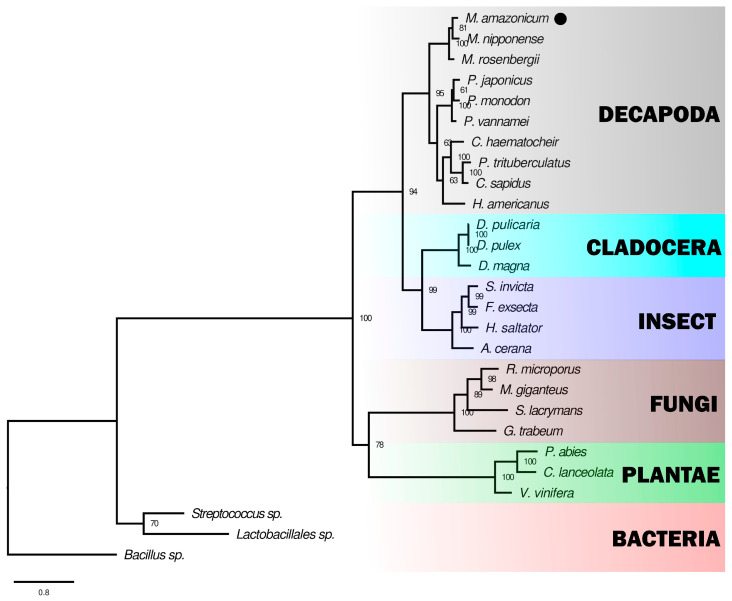
The cladogram illustrates the relationships between the laccase enzyme of *M. amazonicum* (●) and sequences from other biological groups available in NCBI. The cladogram includes decapods (similarity levels: 86.3–58.2%), Cladocera (46.2–44.3%), insects (47.2–46.8%), fungi (29.6–28.7%), Plantae (28.9–27.6%), and bacteria (31.4–22.9%). The cladogram provides maximum support values for the branch comprising invertebrate sequences (100). The cladogram was constructed using maximum likelihood based on the WAG + I + G4 evolutionary model, considering 1000 bootstrap pseudoreplicates.

The 12 sites present in the four copper-binding regions were consistently conserved across crustaceans, along with the C_23_xRxC_27_ and cysteine regions, which accounted for 16 out of 17 sites. The cladogram illustrated a clear structural differentiation within the Decapoda, distinctly separating them from no decapod groups, such as Cladocera and insects. The distinction among laccases from invertebrates, fungi, plants, and bacteria was well-defined (Figure 3), highlighting the low similarity values (<31.4%) between the sequences from non-Metazoan species and *M. amazonicum* (Table 1).

### 3.3. Conformation of the Ma-Lac Protein

**Figure 4:**
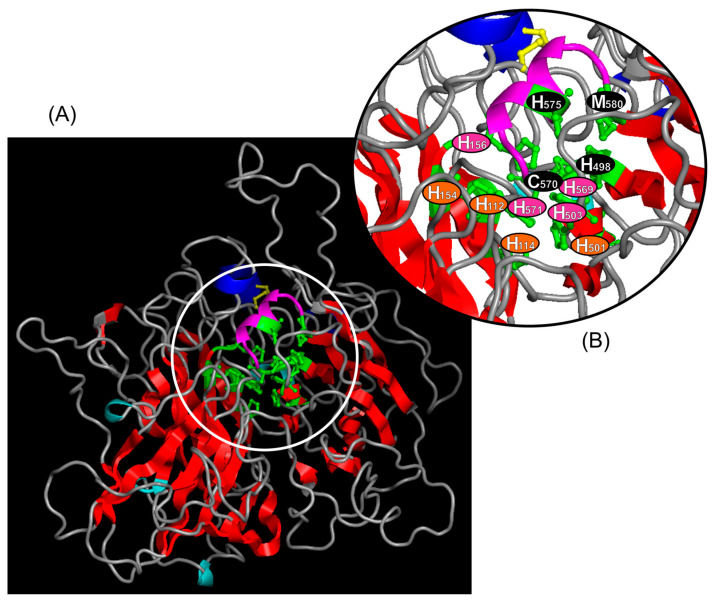
(**A**): The spatial conformation of the laccase characterized in *M. amazonicum* is illustrated, highlighting the following secondary structures: four major α-helices (blue), four minor α-helices (cyan), 31 β-sheets (red), and a disulfide bridge (yellow). (**B**): The 12 copper-binding sites are indicated (green), along with the respective amino acids that compose the TuC1 (black residues), TuC2 (orange residues), and TuC3 (pink residues) centers. The methionine (M_580_), which is typically found in the variable site of laccases from invertebrates, is also shown.

The spatial conformation of the laccase revealed a secondary structure composed of 542 amino acids, featuring four major α-helices, four minor α-helices, 31 β-sheets, 24 internal folds, and a disulfide bond (Figure S1; Figure 4). The conformation model of Ma-Lac was inferred from the sequence of *M. rosenbergii*, exhibiting 85.5% similarity (PDB access: A0A342CJ45.1). A multiple alignment of Ma-Lac with sequences available in the PDB indicated similarity values ranging from 30.9% to 30.2% with *Lentinus* sp. (3X1B_A), *Trametes cinnabarina* (2XYB_A), *L. tigrinus* (2QT6_A), and *T. sanguinea* (5NQ7_A). Despite the low similarity levels in the amino acid sequences, the secondary structures were conserved among the proteins, suggesting a putative conservative functional pathway across different biological groups (Figure S1).

### 3.4. Expression of Ma-Lac

**Figure 5:**
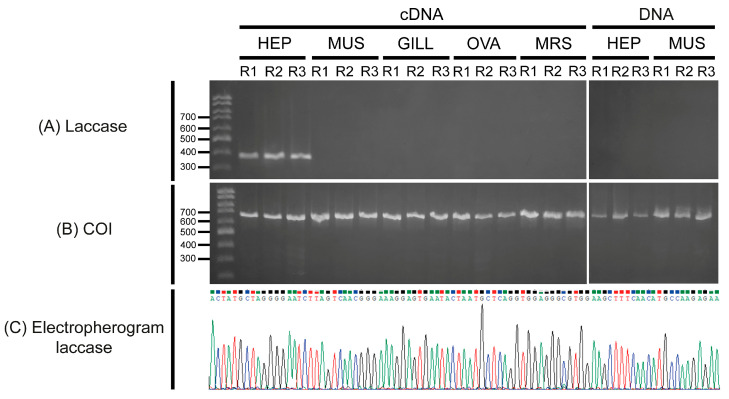
(**A**) _RT_-PCR (cDNA) and PCR (DNA) assays confirming the restricted expression of the laccase gene in the hepatopancreas of adult specimens of *M. amazonicum* based on cDNA analyses. No amplification was observed in the cDNA from the hepatopancreas (HEP), muscle (MUS), gill (GILL), ovary (OVA), or male reproductive system (MRS) tissues, nor in the DNA extracted from the examined tissues (hepatopancreas and muscle). (**B**) The positive control (COI fragment) was successfully amplified in all tested samples. (**C**) The electropherogram of the amplified and sequenced fragment demonstrates the high quality of the final sequence. R: replicate.

The expression of the Ma-Lac encoding gene was analyzed in different tissues of *M. amazonicum* through cDNA analyses using specific primers designed based on the consensus sequence. Expression profiles indicated that Ma-Lac was only expressed in the hepatopancreas, with no expression detected in other tissues, including muscle, gills, ovary, and male reproductive system. In contrast, the positive control (COI gene) was successfully expressed in all tissue replicates. Amplification attempts using DNA templates from both the hepatopancreas and muscle yielded successful results only for the control gene, while Ma-Lac did not amplify (Figure 5). The fragment of the gene used in the expression test was sequenced, revealing a 100% similarity with the consensus sequence (accession number PQ319833.1), confirming that this result was not due to nonspecific amplification.

**Figure 6:**
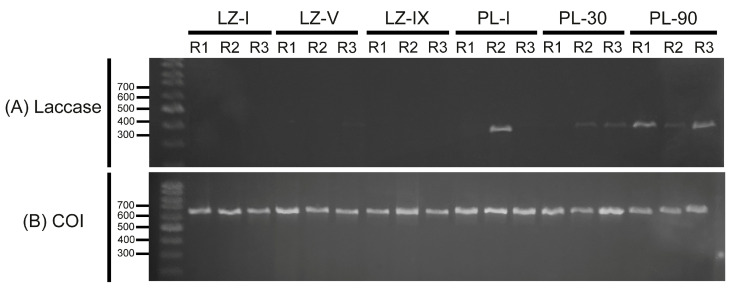
(**A**) _RT_-PCR (cDNA) assays of the *laccase* enzyme across various stages of ontogenetic development in *M. amazonicum* indicated no amplification in the larval stages LZ-I, LZ-V, and LZ-IX. An increase in expression was observed starting from the post-larvae 30 (PL-30) stage, with the highest expression levels recorded at PL-90. (**B**) Amplification profiles for the positive control (COI gene) were detected in all tested samples. R: replicate.

The expression of the Ma-Lac enzyme did not exhibit distinct bands across the various ontogenetic development stages. However, relatively weak bands were observed in post-larvae 30 (PL-30), while post-larvae 90 (PL-90) displayed higher levels of expression comparable to those recorded in the hepatopancreas of adult specimens (Figure 6). The control gene was expressed in all tested samples (larvae zoea I [LZ-I], larvae zoea V [LZ-V], larvae zoea IX [LZ-IX], post-larvae I [PL-I], post-larvae 30 [PL-30], and post-larvae 90 [PL-90]).

## 4. Discussion

The Amazon River prawn is an omnivorous species that primarily feeds on plant-based resources. Recent findings have revealed the expression of enzymes related to cellulose degradation in its functional genome, suggesting that the availability of these enzymes may play a crucial role in enabling the prawn to utilize cellulose and hemicellulose from crude vegetable fibers as energy sources. In this study, we identified the *laccase-encoding* gene (Ma-Lac) in the transcriptome of the hepatopancreas of *M. amazonicum*, with exclusive expression in this tissue. In silico protein characterization confirmed the presence of all conserved sites, including those observed only in laccase-like enzymes of animal species.

Laccases are a member of the multicopper oxidase enzyme family, classified under phenoloxidases, and are distinguished by the presence of Cu-oxidase domains. These domains are crucial for initiating lignin depolymerization processes [22]. In *M. amazonicum*, three conserved multicopper oxidase (3dMCO) domains were identified, consistent with the copper oxidases found across a wide range of species, including fungi, plants, insects, crustaceans, as well as some bacteria and archaea [27,29,53,54,55,56]. These enzymes are known to participate in oxidation reactions involving various phenols and diamines. Additionally, two-domain multicopper oxidases (2dMCO) have been found in certain bacteria and fungi [57,58], while six-domain variants (6dMCO) are present in mammalian proteins, particularly in ceruloplasmin [59].

Similar to other biological groups exhibiting laccase enzymes, the *M. amazonicum* laccase contains 12 conserved residues related to the mononuclear (T1Cu) and trinuclear copper (T2Cu/T3Cu) centers (Figure 1). In laccases with a 3-domain multicopper oxidase (3dMCO) configuration, the mononuclear center is located in domain III, which is responsible for binding copper ions, while the trinuclear center forms at the interface between domains I and III, where three copper ions attach [25,60]. The enzyme’s oxidation activity initiates at the T1Cu center, where an electron is transferred to the copper substrate and then passed to the T2Cu/T3Cu center, with oxygen acting as the final electron acceptor, reducing to a water molecule [61]. Due to the interaction between copper ions and their binding centers, laccases typically exhibit several β-sheets in their conformation, which help ensure a stable molecular structure [62] (Figure 4; Figure S1).

Laccases are classified into three categories based on their reduction potentials (E°), which are influenced by the amino acid residue present at the fourth position of the active T1Cu site (H, C, H, x) [25,26]. In *M. amazonicum*, methionine was identified at this position (Figure 1), indicating that Ma-Lac possesses a low reduction potential (<+460 mV). This pattern is consistent with laccases found in other arthropods [27,28,29,53,63], plants, certain bacteria, and mitosporic fungi [53,64,65,66,67]. Laccases with leucine at this site exhibit medium E° values (460–710 mV), typically observed in ascomycetes, deuteromycetes, and some basidiomycetes [65,68,69]. In contrast, laccases with phenylalanine have the highest E° values (>+710 mV), commonly found in white-rot basidiomycete fungi [60,70,71], which enhances their ability to oxidize phenolic and polyphenolic compounds in lignin.

The homology analysis of the *M. amazonicum* laccase (Ma-Lac) showed high similarity with laccase proteins from *M. nipponense* and *M. rosenbergii* (~85%), followed by sequences from penaeids, crabs, and crayfish (64.1–58.2%). In *L. vannamei*, two isoforms of laccase have been identified, Lv-Lac1 and Lv-Lac2, which are associated with immune defense against pathogens and responses to oxidative stress [27,28]. Similarly, in insects, two isoforms are present, with Lac-1 exhibiting digestive activity alongside various biological functions [53,72]. In contrast, *M. amazonicum* has only a single laccase isoform, expressed solely in the hepatopancreas, while in *L. vannamei*, the enzyme is active in multiple tissues, including the eyestalk, intestine, gills, and heart.

Expression of Ma-Lac during the ontogenetic development of *M. amazonicum* was not uniform, with consistent expression starting from the PL-90 stage, a pattern observed in other decapods. This suggests that *M. amazonicum* may employ distinct strategies for nutritional resource utilization throughout its development [73,74,75]. The findings are comparable to those in the herbivorous crab *C. haematocheir*, which also expresses a single laccase isoform primarily in the hepatopancreas, with activity over lignin residues [29]. This suggests potential evolutionary adaptation to similar dietary or physiological demands in these species.

The Ma-Lac enzyme characterized in *M. amazonicum* was not amplified when DNA from hepatopancreas and muscle tissues was used as a template, which suggests that this enzyme may not be expressed endogenously at the DNA level in the tissues tested. However, this result might be due to the fact that the primers (Lac-F and Lac-R) were designed based on transcript data from the transcriptome, and their performance might have been influenced by unknown factors that were not detectable through bioinformatics analysis.

Despite this, Ma-Lac shares high homology with other crustacean sequences, including the characteristic CxRxC region, a conserved domain specific to arthropod laccases, which is absent in fungi, plants, and bacteria [63,76]. This points towards an endogenous expression pattern in *M. amazonicum*, akin to findings in the crab *C. haematocheir*, where laccase was confirmed to be endogenous [29]. The cladogram also supports this hypothesis, showing that laccase enzymes from decapods form a distinct and well-supported evolutionary cluster (Figure 3), with Ma-Lac displaying low similarity compared to noninvertebrates. These data reinforce the idea that the synthesis of Ma-Lac is not due to symbiotic relationships but is an inherent feature of the species (Table 1).

Recent studies have identified the endogenous Endo-β-1,4-glucanase and Endo-β-1,4-mannanase enzymes in the transcriptome of *M. amazonicum*, further indicating the prawn’s capacity to digest plant fibers [16,17]. Queiroz et al. [17] observed increased Endo-β-1,4-glucanase expression in response to a cellulose-enriched diet, alongside the expression of both glucanase and mannanase at developmental stages PL-30 and PL-90. In this study, laccase expression was similarly detected at these stages, suggesting that *M. amazonicum* possesses enzymes for digesting lignin, hemicellulose, and cellulose during these stages. This developmental enzyme expression pattern aligns with findings in the crab *C. haematocheir*, whose laccase enzyme was activated by plant-based diets [29].

These results support *M. amazonicum* ability to utilize plant-derived carbon, specifically in breaking down cellulose in its diet, demonstrating its feeding flexibility and adaptability to plant matter [29]. The presence of a phenoloxidase enzyme further reinforces the prawn’s capacity to incorporate plant-based materials [8,12,17]. However, while the study confirmed the presence and in silico characterization of laccase, further experimental research is needed to establish its precise functional role. In decapods, laccase might either aid in accessing dietary cellulose and hemicellulose [29] or serve functions related to immune responses [27,28]. More research will help clarify these potential roles.

To efficiently utilize the nutrients in plant-based diets, digestive enzymes in organisms like *M. amazonicum* must target key components of raw plant fibers: cellulose, hemicellulose, and lignin. In certain herbivorous crustaceans, such as Isopoda and Amphipoda, hemocyanins like phenoloxidases take on this role, breaking down lignin to enhance accessibility to other plant fibers. In species where laccase or prephenoloxidases are absent (e.g., *Limnoria quadripunctata*), hemocyanins play a role in increasing the porosity of lignocellulosic fibers, facilitating cellulose degradation [77,78,79]. This suggests that distinct adaptive strategies have evolved in various zoological groups to increase cellulose accessibility in plant-based diets.

In light of recent nutrigenomic studies on carbohydrate metabolism in *M. amazonicum*, it appears that this species relies on phenoloxidase-like enzymes, such as the laccase characterized in this study. These enzymes enable the prawn to metabolize cellulose and hemicellulose as energy sources, further supporting its ability to thrive on diets rich in plant fibers. This adaptation highlights the evolutionary mechanisms crustaceans have developed to digest complex plant materials and extract necessary nutrients [80].

## 5. Conclusions

In this study, we conducted the first in silico characterization of the enzyme laccase in the transcriptome of palaemonid crustaceans, offering new insights into the nutritional aspects of Amazon River shrimp. These findings have the potential to enhance technological processes related to the cultivation of Amazon River shrimp, particularly by developing more efficient diets tailored to the species’ specific nutritional requirements. This could result in a more cost-effective approach and improved animal performance.

## Figures and Tables

**Table 1:** A description of the transcripts identified in the transcriptome databases of the hepatopancreas of *M. amazonicum* is provided, highlighting the sequence used for the characterization of the laccase enzyme (*). The sequences available in NCBI, utilized for constructing alignments and the cladogram, are listed, indicating fragment sizes and similarity levels when compared to the laccase registered in *M. amazonicum*, along with the accession codes and references. Species marked with two asterisks (**) indicate published data confirming the endogenous presence of the gene in those respective species. Abbreviations used include Nt (nucleotide size), aa (protein size), and % aa (similarity between Ma-Lac and the laccases of other species).

Species	Taxon	Nt	Aa	% Aa	Access NCBI	Reference
*Macrobrachium amazonicum* *	Metazoa: Crustacea	2570	609	-	PQ319832.1	Present study
*M. amazonicum*	Metazoa: Crustacea	2207	609	99.7	PQ498382.1	Present study
*M. amazonicum*	Metazoa: Crustacea	2607	609	99.7	PQ498383.1	Present study
*M. amazonicum*	Metazoa: Crustacea	2530	609	99.7	PQ498384.1	Present study
*Macrobrachium nipponense*	Metazoa: Crustacea	2225	605	86.3	XP_064108161.1	Unpublished
*Macrobrachium rosenbergii*	Metazoa: Crustacea	2409	609	85.5	AJG06864.1	Unpublished
*Penaeus japonicus*	Metazoa: Crustacea	2311	610	64.1	XP_042889082.1	Unpublished
*Penaeus vannamei* **	Metazoa: Crustacea	2326	610	64.3	QLP89093.1	Chen et al. [28]
*Penaeus monodon*	Metazoa: Crustacea	2490	610	62.7	XP_037783279.1	Unpublished
*Chiromantes haematocheir* **	Metazoa: Crustacea	2465	613	62.2	BCO16709.1	Miyake et al. [29]
*Portunus trituberculatus*	Metazoa: Crustacea	92,768	849	61.9	MPC11944.1	Unpublished
*Homarus americanus*	Metazoa: Crustacea	2408	609	58.2	XP_042217468.1	Unpublished
*Callinectes sapidus*	Metazoa: Crustacea	1969	566	63.1	ALS03818.1	Unpublished
*Daphnia pulicaria*	Metazoa: Crustacea	3474	707	46.2	XP_046632502.1	Unpublished
*Daphnia pulex*	Metazoa: Crustacea	3419	707	45.9	XP_046453699.1	Unpublished
*Daphnia magna*	Metazoa: Crustacea	1,051,620	717	44.3	KZS15188.1	Unpublished
*Solenopsis invicta*	Metazoa: Hexapoda	4851	724	47.1	XP_025992935.1	Unpublished
*Formica exsecta*	Metazoa: Hexapoda	2595	773	47.2	XP_029665984.1	Unpublished
*Apis cerana*	Metazoa: Hexapoda	2883	726	46.8	XP_016917075.1	Unpublished
*Harpegnathos saltator*	Metazoa: Hexapoda	292,706	846	47	EFN87217.1	Unpublished
*Rigidoporus microporus* [white]	Fungi: Basidiomycota	2201	518	29.1	AAO38869.1	Unpublished
*Meripilus giganteus* [white]	Fungi: Basidiomycota	2214	516	29	CBV46340.1	Unpublished
*Serpula lacrymans* [brown]	Fungi: Basidiomycota	1566	521	29.6	XP_007321217.1	Eastwood et al. [43]
*Gloeophyllum trabeum* [brown]	Fungi: Basidiomycota	1629	542	28.7	XP_007867384.1	Floudas et al. [44]
*Picea abies*	Viridiplantae: Embryophyta	1955	570	27.9	AFV52380.1	Koutaniemi et al. [45]
*Vitis vinifera*	Viridiplantae: Embryophyta	565,974	597	27.6	RVW56841.1	Roach et al. [46]
*Cunninghamia lanceolata*	Viridiplantae: Embryophyta	3345	570	28.9	QBC40966.1	Unpublished
*Bacillus* sp.	Bacteria: Bacilli	1539	512	22.9	ASO96841.1	Unpublished
*Streptococcus* sp.	Bacteria: Bacilli	1443	480	25.1	WP_044685747.1	Grossmann et al. [47]
*Lactobacillales* sp.	Bacteria: Bacilli	1530	509	31.4	WP_134658255.1	Page et al. [48]

## Data Availability

The data generated in this study has been deposited at the National Center for Biotechnology Information (NCBI) under the accession numbers PQ319832.1, PQ319833.1, PQ498382.1, PQ498383.1, PQ498384.1. The sequences are currently private and will be made public with the publication of this study.

## Supplementary Materials

The following supporting information can be downloaded at: https://www.mdpi.com/article/10.3390/genes15111416/s1, Table S1. Top hits generated using the BLASTp algorithm, comparing the laccase sequence from *M. amazonicum* against the NCBI database. This table highlights the 40 hits with the highest similarity, exclusively composed of decapod crustaceans (prawn, crayfish, and crabs), with similarity levels ranging from 86.3% to 51.4%. Species highlighted in gray have published studies confirming the endogenous expression of the enzyme within this group. accessed on 06 March 2024. Figure S1. Multiple alignments of the laccase from *M. amazonicum* and other proteins available in the Protein Data Bank (PDB). This figure reveals a comparative analysis of the predicted secondary structures, which comprise four major α-helices, four minor α-helices, 31 β-sheets, 24 internal fold bridges (TT), and one disulfide bridge (C-C). These structures are highly conserved across different biological groups. The highly, semi-, and weakly conserved amino acid sequences are indicated in red, yellow, and white, respectively. The species used in the alignments include *Lentinus* sp. (3X1B_A), *Trametes cinnabarina* (2XYB_A), *L. tigrinus* (2QT6_A), and *T. sanguinea* (5NQ7_A), displaying similarity levels between 30.9% and 30.2%.
