# Supplementary figures and images for: In Silico Identification of the Laccase-Encoding Gene in the Transcriptome of the Amazon River Prawn Macrobrachium amazonicum (Heller, 1862)
Source: Genes (Basel). 2024 Oct 31;15(11):1416. doi: 10.3390/genes15111416 (PMC11593427; doi:10.3390/genes15111416)

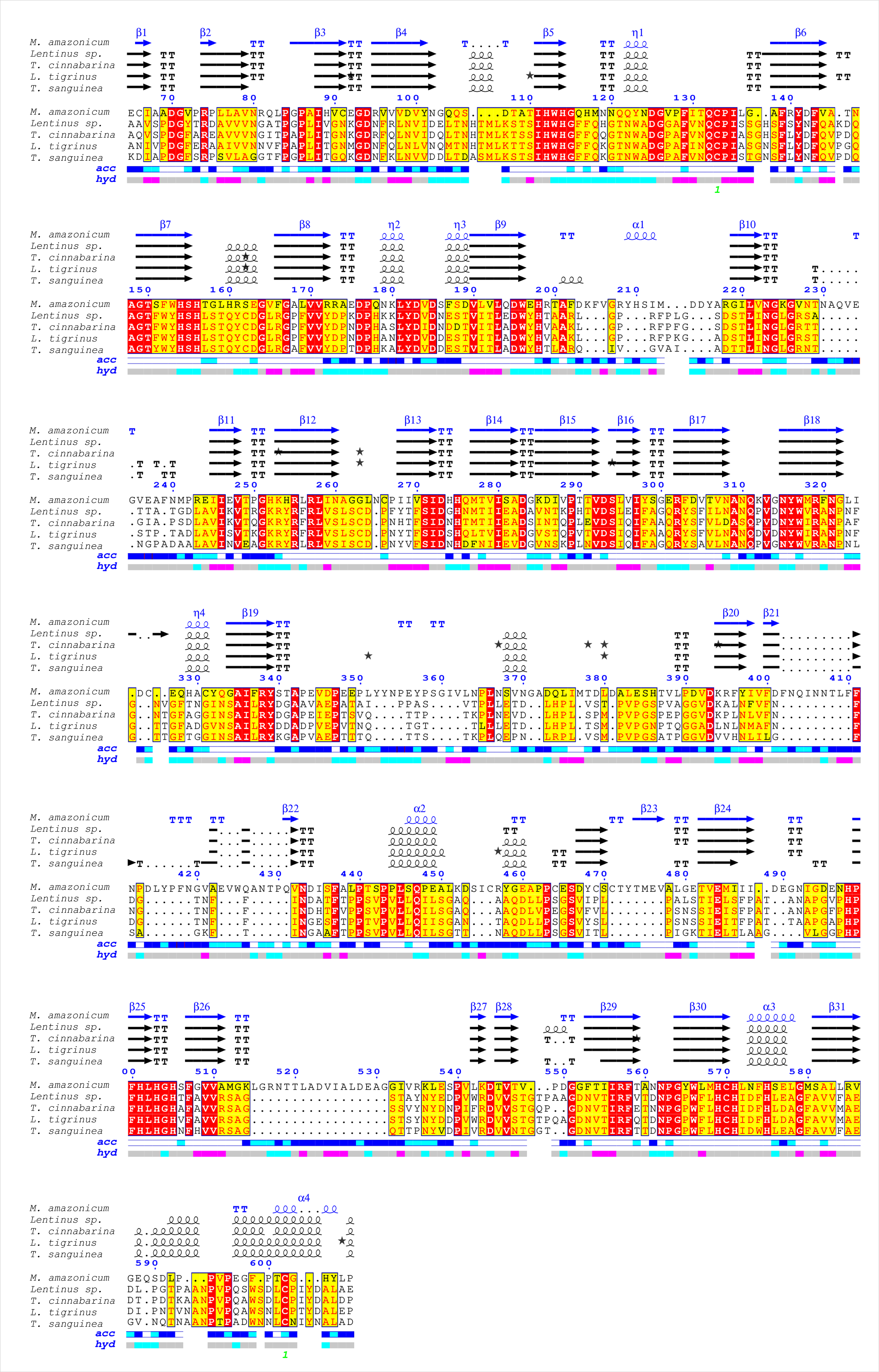

Supplement: Supplementary file 1 [file genes-15-01416-s001.zip › Figure S1.tif]
